# Isolation, expansion and characterization of porcine urinary bladder smooth muscle cells for tissue engineering

**DOI:** 10.1186/s12575-016-0047-9

**Published:** 2016-08-12

**Authors:** Marta Pokrywczynska, Daria Balcerczyk, Arkadiusz Jundzill, Maciej Gagat, Monika Czapiewska, Tomasz Kloskowski, Maciej Nowacki, Agata M. Gastecka, Magdalena Bodnar, Alina Grzanka, Andrzej Marszalek, Tomasz Drewa

**Affiliations:** 1 Department of Regenerative Medicine, Nicolaus Copernicus University in Torun, Ludwik Rydygier Medical College in Bydgoszcz, Chair of Urology, Karlowicza 24 Street, 85-092 Bydgoszcz, Poland; 2 Chair of Histology and Embryology, Nicolaus Copernicus University in Torun, Ludwik Rydygier Medical College, Bydgoszcz, Poland; 3 Chair of Surgical Oncology, Nicolaus Copernicus University in Torun, Ludwik Rydygier Medical College, Bydgoszcz, Poland; 4 Department of Clinical Pathomorphology, Nicolaus Copernicus University in Torun, Ludwik Rydygier Medical College, Bydgoszcz, Poland; 5 Department of Pathology, Poznan University of Medical Sciences, Poznan, Poland; 6 Department of Urology, Nicolaus Copernicus Hospital, Torun, Poland

**Keywords:** Smooth muscle cells, Isolation, Primary culture, Tissue engineering

## Abstract

**Background:**

A key requirements for therapy utilizing the tissue engineering methodologies is use of techniques which have the capability to yield a high number of cells, from small tissue biopsy in a relatively short time. Up to date there was no optimal methods of isolation and expansion of urinary bladder smooth muscle cells (UB-SMCs). The aim of this study was to compare isolation and expansion techniques of UB-SMCs to select the most repeatable and efficient one.

**Method:**

Five protocols of porcine UB- SMCs isolation including enzymatic and explant techniques and three expansion techniques were compared. Isolation effectiveness was evaluated using trypan blue assay. Cell phenotype was confirmed by immunofluorescence staining. Proliferation rate was analyzed using MTT and X- Celligence system. Cellular senescence was assessed measuring β*-*galactosidase activity.

**Results:**

Enzymatic methods using collagenase with dispase (method I) or collagenase only (method III) allowed to isolate much larger number of cells than the methods using trypsin with collagenase (method II) and collagenase after digestion with trypsin (method IV). The success rate of establishment of primary culture was the highest when the isolated cells were cultured in the Smooth muscle Growth Medium-2 (SmGM-2). Expression of the smooth muscle markers- alpha smooth muscle actin and smoothelin was the highest for cells isolated by enzymatic method I and cultured in SmGM-2. There was no significant signs of cell senescence until the 8th passage.

**Conclusion:**

The most efficient method of establishment of porcine UB-SMCs culture is enzymatic digestion of urinary bladder tissue with collagenase and dispase and culture of isolated cells in SmGM-2. This method was up to 10 times more efficient than other methods used for isolation and culture of UB-SMCs. This is an easy and consistent method for obtaining high numbers of urinary bladder smooth muscle cells.

## Background

Augmentation cystoplasty with autologous intestine remains the gold standard of surgical treatment for many patients with low capacity, poorly compliant or refractory overactive urinary bladder. However, because intestinal tissue is functionally different from the urinary bladder tissue its use is burdened by multiple complications [1].

Tissue engineering give a possibility to create an urinary bladder de novo using biomaterials and patients’ cells [2]. Promising results of urinary bladder augmentation with the tissue engineered grafts in children with neurogenic bladder caused by myelomeningocele opened a new era in reconstructive urology [3]. Multiple experimental studies indicated that urinary bladders augmented with cell seeded grafts heal by regeneration in contrast to urinary bladders augmented with unseeded grafts which heals by repair with formation of scar tissue [4–8]. Smooth muscle cells seeded on grafts play multiple functions: seal highly porous grafts and protect by urine leakage, prevent graft fibrosis, enhance vascularization and trigger smooth muscle regeneration. One of the most important factor that affect an urinary bladder regeneration is number of cells used for graft seeding. It is widely known that high numbers of cells are required for efficacious regeneration. Therefore critical is use of isolation and expansion techniques which have the capability to yield a high number of cells, from small urinary bladder biopsy in a relatively short time. Several techniques of establishment of primary cultures of urinary bladder smooth muscle cells have been described in the literature [9–14]. However up to now, the optimal protocol for establishment of UB-SMCs culture is unknown. Successful establishment of UB-SMCs culture is extremely difficult and the success rate is dependent on numerous factors including methods of cell isolation (enzymatic digestion or explant culture, enzyme type and concentration, digestion time and temperature) and cultivation (density of cell seeding, type of culture medium). Establishment of UB-SMCs culture for tissue engineering is even more complicated because cells are usually isolated from a small biopsy and have to be expanded to high numbers.

The purpose of this study was to compare the isolation and expansions techniques of UB-SMCs to select the most repeatable and efficient for use in tissue engineering.

## Methods

### Animals

The study was carried out with the permission of the Local Ethics Committee (no. 32/2013). Thirteen urinary bladders of the male domestic pigs with an average body weight of 120 kg were obtained from the local abattoir. Organs immediately following resection were placed in the containers filled with Dulbecco’s Modified Eagle Medium/ Ham’s F12 (DMEM/Ham’s F12, HyClone, USA) supplemented with antibiotics: penicillin (100U/ml, PAA, Austria), streptomycin (100 μg/ml, PAA, Austria), amphotericin B (5 μg/ml, Sigma, Germany) and transported to the laboratory.

### Tissue processing

**Fig. 1:**
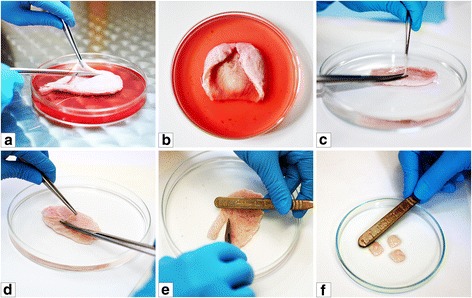
Preparation of the fragments of smooth muscle layer for isolation of urinary bladder smooth muscle cells (**a**-**f**)

The tissues were proceeded within 2 h following surgical removal. All procedures were carried out in a laminar flow hood, using sterile techniques. Urinary bladder, immediately upon arrival was placed in a large Petri dish (15 cm diameter) containing DMEM/Ham’s F-12 supplemented with antibiotics. The adjacent connective and fatty tissues were removed. Urinary bladder was opened longitudinally and bladder neck including trigone and urethra were resected. Mucosa/submucosa and serosa were removed from the remaining bladder wall using a small surgical scissors (Fig. 1). Complete removal of the mucosa/submucosa and serosa was confirmed by histological and immunohistochemicalstainings. Detrusor smooth muscle layer was cut into 1 cm^2^ pieces. Tissue fragments were then washed five times with Phosphate Buffered Saline (PBS, Sigma, Germany) and transferred to DMEM/Ham’s F12 supplemented with penicillin/streptomycin, amphotericin B and metronidazole (100 μg/ml, Polpharma, Poland).

### Histological and immunohistochemical stainings

In order to confirm removal of the mucosa/submucosa and serosa, the fragments of the smooth muscle layer (1 cm^2^) were fixed for 48 h in 10 % buffered formalin, embedded in paraffin, sectioned at 4 μm tick sections and stained routinely with hematoxylin and eosin. To identify urothelial cells, immunohistochemical staining with monoclonal antibody against the p63 protein (dilution of the manufacturer, Roche, Germany) was performed. Presence of the smooth muscle cells was confirmed using a mouse monoclonal antibody against α-smooth muscle actin (anti- alpha smooth muscle actin [1A4] antibody, dilution of the manufacturer, Abcam, UK). Antigen- primary antibody complexes were visualized by use of a secondary antibody conjugated to horseradish peroxidase (EnVisionTM FLEX anti Mouse/Rabit HRP; Dako, Denmark).

### Urinary bladder smooth muscle cells isolation

Five previously described protocols including four enzymatic digestion methods (methods I- IV) and one explant technique (method V) with slight modifications (Table 1) were used for isolation of UB- SMCs [9–12]. In order to exclude differences in isolation yields depending on the inter individual variability, all methods were compared using samples obtained from the same donor. A total of 180 enzymatic isolations (9 bladders × 4 methods × 5 samples) and 27 explants cultures were performed.

**Table 1:** Protocols used for establishment of urinary bladder smooth muscle cells culture

Method	Source article	Modified element	Originally	After modification
I	McCoy 2013 [9]	Tissue source	Human	Pig
		Quantity of the tissue	1g	1 cm^2^
		Digestion solution	Collagenase IV (450U/ml), Dispase II, CaCl_2_ 5mM	Collagenase II (0.15%, 275U/mg), Dispase II (0.2%, 175U/mg), CaCl_2_ 5mM, HBSS^a^
		Volume of the digestion solution	40ml	5ml
		Digestion conditions	1h, 37°C	1.5h, 37°C
		Seeding density	All cells/300–350cm^2^	20 000 cells/cm^2^
		Growth media	DMEM High glucose^b^, FBS^c^ (10%), Gentamycin (5μg/ml)	A: DMEM High glucose^b^, FBS^c^ Pan-Biotech (10%), Gentamycin (100μg/ml), Amphotericin B (5μg/ml), Penicillin (100U/ml), Streptomycin (100μg/ml)
				B: DMEM High glucose^b^, FBS^c^ Sigma (10%), Gentamycin (100μg/ml), Amphotericin B (5μg/ml), Penicillin (100U/ml), Streptomycin (100μg/ml)
				C: SmGM-2
II	Cheng et al. 2011 [10]	Tissue source	Pig	Pig
		Quantity of the tissue	No data	1 cm^2^
		Digestion solution	Trypsin-EDTA (0,25%) Collagenase II (0.15%)	Trypsin (0.25%), EDTA (0.02%), Collagenase II (0.15%, 275U/mg), CaCl_2_ 5mM
		Volume of the digestion solution	No data	5ml
		Digestion conditions	30min., 37°C	1h, 37°C
		Seeding density	10^6^ cells/well (48-well plate)	20 000 cells/cm^2^
		Growth media	RPMI 1640^d^, FBS^c^ (10%)	A, B, C
III	Lau et al. 1996 [11]	Tissue source	Rabbit	Pig
		Quantity of the tissue	Whole urinary bladder muscle layer	1 cm^2^
		Digestion solution	Collagenase I (0.1%), SFNM^e^	Collagenase II (0.1%, 275U/mg), CaCl_2_ 5mM, DMEM High glucose^b^
		Volume of the digestion solution	20ml	5ml
		Digestion conditions	~16h, 37°C	~16h, 37°C
		Seeding density	10^5^ cells/35mm culture dish	20 000 cells/cm^2^
		Growth media	SFNM^e^ (M199, Sodium bicarbonate (2.2g/l), ×100 BME vitamins (10ml/l), ×50 BME amino acids (20 ml/1), Fungizone (1 ml/l),Penicilin/Streptomycin solution (100 U/ml Penicillin G and 100pg/ml Streptomycin)), FBS^c^(10%)	A, B, C
IV	Ma et al. 2002 [12]	Tissue source	Rat	Pig
		Quantity of the tissue	Whole urinary bladder muscle layer	1 cm^2^
		Digestion solution	1. trypsin (0.2%) 2. Collagenase (0,1%), RPMI 1640^d^	1. trypsin (0.2%) 2. Collagenase II (0.1%, 275U/mg) CaCl_2_ 5mM, DMEM High glucose^b^
		Volume of the digestion solution	No data	5ml
		Digestion conditions	1. 30min., 37°C 2. 30min., 37°C	1. 30min., 37°C 2. 1h, 37°C
		Seeding density	No data	20 000 cells/cm^2^
		Growth media	RPMI 1640^d^, FCS (10%)	A, B, C
V	McCoy 2013 [9]	Tissue source	Human	Pig
		Number of explants	20–25 tissue explants (1mm diameter)	explants from 1 cm^2^ of tissue (1mm diameter)
		Culture dish	100 mm tissue culture Petri dish	60 mm tissue culture Petri dish
		Growth media	DMEM High glucose^b^, FBS^c^ (10%), Gentamicin (5μg/ml)	A, B, C

^a^ *HBSS* Hank’s Balanced Salt Solution

^b^ *DMEM High glucose* Dulbecco’s Modified Eagle Medium with high glucose

^c^ *FBS* Fetal Bovine Serum

^d^ *RPMI 1640* Roswell Park Memorial Institute medium 1640

^e^ *SFNM* Serum-Free Nutrient Medium

### Methods I- III

Fragments of the smooth muscle layer (1 cm^2^) were minced into small pieces (1 mm^2^) and digested in collagenase II and dispase II (method I), collagenase II and trypsin (method II) and collagenase II (method III) for 1.5, 1 and 16 h respectively (Table 1). After digestion time the enzymes were neutralized by addition of an equal volume of medium containing FBS. The resulting suspensions were filtered through 100 μm nylon cell strainers (BD, USA) and centrifuged at 1500xg for 5 min. Cell pellet was resuspended in culture medium. The number of isolated cells was estimated using trypan blue exclusion test.

#### Method IV

Fragments of the smooth muscle layer (1 cm^2^) were incubated in trypsin for 30 min (Table 1). Next, the fragments were transferred to the Petri dish where using the blunt side of a scalpel they were swabbed to get rid of any residue mucosa/submucosa and serosa. Then fragments were minced into small pieces and incubated for 1 h in collagenase type II. Enzymatic digestion was stopped by the addition of medium containing FBS. Resulting suspension was centrifuged (2 min., 250xg), the supernatant containing cells was collected, and the pellet was resuspended in medium. Centrifugation (2 min., 150xg) and supernatant collection procedures were repeated. Undigested tissue sediment was discarded, and the collected cell suspensions were mixed and centrifuged (5 min., 1500xg). Finally, cell pellet was resuspended in growth medium and number of cells was estimated with trypan blue assay.

### Method V

Tissue fragments (1 cm^2^) were cut into small pieces and placed on the bottom of the 60 mm culture dish. Petri dish with explants was left open for 10- 15 min. in a laminar flow cabinet in order to fix the tissue. Next the culture medium was added cautiously on the surface of the dish, so as not to disturb the attached fragments of muscle layer. Finally the dish was placed in an incubator (37 °C, 5 % CO_2_). First medium change was made on the 3rd day of culture and at the same time the detached tissue fragments were removed. Cultures were grown until the formation of large, confluent colonies. Growth media were changed every 2–3 days.

### Cell culture and media

To select the best method of establishment of primary culture of UB-SMCs a total of 135 cell cultures were established (5 isolation protocols × 3 media × 9 isolations).

Isolated cells were seeded at a density of 2 × 10^4^ cells/cm^2^. Three different growth media were compared. Two media (A and B) consisted of DMEM HG supplemented with 100U/ml penicillin, 100 μg/ml streptomycin, 5 μg/ml amphotericin B, 100 μg/ml gentamycin and one of the two sera: 10 % FBS Good (Medium A; Pan-Biotech, Germany) or FBS Sigma (Medium B; Sigma, Germany). Third medium was a Smooth muscle Growth Medium, SmGM-2 (Medium C; Lonza. Germany). Cells were cultured at 37 °C in 5 CO_2_ and 95 % humidity. Growth medium was changed every 2–3 days. Cell morphology and growth were evaluated under inverted light microscope.

### Success rate of primary culture

Cell cultures that have reached 70- 90 % confluence and showed morphology typical for smooth muscle cells were considered as a successful. Any irregularities such as changes in morphology or detachment of the cells were regarded as a failure. Success rate was calculated using the formula:

$$ S=\frac{n}{N}\times 100\% $$

- S - success rate [%],
- n - number of successfully established cultures,
- N - total number of cultures.

#### Analysis of cell phenotype by immunofluorescence

To analyze the phenotype of cells in primary cultures immunofluorescent staining of anti- alpha smooth muscle actin (α-SMA) and anti- smoothelin were performed. Isolated cells were seeded into 24-well cell culture plate with glass coverslips. In the 5th day after the establishment of the primary culture, cells were fixed by 15 min. incubation in 2 % paraformaldehyde (Sigma, Germany). Non-specific antibody binding sites were blocked by incubation with 1 % bovine serum albumin (BSA, Sigma, Germany) in PBS. As primary antibodies murine monoclonal anti- α-smooth muscle actin antibody (dilution 1:100, anti-alpha smooth muscle actin [1A4] antibody, Abcam, UK,) and murine monoclonal anti-smoothelin antibody (dilution 1:100, Anti-Smoothelin antibody [R4A], Abcam, UK) were used. Cultures were incubated with primary antibodies for 1.5 h. Thereafter, cells were incubated (room temperature, 1 h) with the secondary antibody conjugated with a fluorescent dye (dilution 1:100, anti-mouse Alexa Fluor 488, Life Technologies, Germany). Nuclei were stained by 10 min. incubation with DAPI (dilution 1:20.000, Sigma, Germany). After washing with PBS, mounting medium Aqua-Poly/Mount (Polysciences, Inc., USA) was applied. Microscopic slides were analyzed using a confocal microscope (Nikon Eclipse Ti-U, Japan).

Measurements of the fluorescence intensity were performed using the EZ-C1 3.90 FreeViewer software (Nikon, Japan). Fluorescence intensity of individual cells, cells surface and fluorescence intensity of background were measured. Resulting data were used to calculate relative cell fluorescence (RCF):

$$ RCF=\left(cs\times f{i}_c\right)-\left(cs\times f{i}_b\right) $$

- RCF - relative cell fluorescence,
- cs - cell surface [μm^2^],
- fi_c_ - cell fluorescence intensity,
- fi_b_ - background fluorescence intensity.

### Analysis of cell phenotype by flow cytometry

Cells isolated using method I and cultured in SmGM-2 medium were additionally analyzed using flow cytometry. Three independent experiments from three different isolations were performed. To confirm efficiency of isolation the alpha-Smooth Muscle Actin Antibody (α-SMA; Novus Biologicals, USA) labeled with PE was used. In order to exclude unspecific binding of antibody cells labeled with PE Mouse IgG2a, k Isotype Control, (BD Pharmingen, USA) were used. Approximately 0.5x10^6^ cells were used for analysis. Cells were washed twice in PBS, centrifuged for 5 min at 700 × g and suspended in 200 μL of Staining Buffer (BD Biosciences, USA) containing 2 FBS and 0.09 % sodium azide. Concentration of α-SMA antibody and isotype control were 1 μl and 4 μl per 1mln of cells respectively. Cells were then incubated for 30 min in dark at 4 °C. After incubation cells were washed twice with Staining Buffer and analyzed with the use of FACSCanto II flow cytometer (BD Biosciences, USA).

### Selection of the most effective method of isolation and establishment of primary culture of UB-SMCs

To select the most effective method of isolation and establishment of primary culture of UB-SMCs, parameters such as: number of isolated cells, cell morphology, success rate of the cultures and cells phenotype were compared. UB-SMCs obtained using this method were further characterized in vitro.

### Cell proliferation and population doubling time

#### Trypan blue assay

Cells from the 1st passage were seeded on 24-well plates in density of 2 × 10^4^ cells/cm^2^. For seven consecutive days cells were detached from the wells (4 wells every day) and their number was determined by trypan blue exclusion test. Growth curve was drawn based on the number of harvested cells. Using number of cells obtained in the following days of logarithmic growth phase, population doubling time (PDT) was calculated:

$$ PDT=\frac{T}{3.32\left( \log {X}_e- \log {X}_b\right)} $$

- T - duration of logarithmic growth phase,
- X_b_ - number of cells collected at the beginning of the logarithmic growth phase,
- X_e_ - number of cells collected at the end of the logarithmic growth phase.

### MTT assay

Cells from the 1st passage were seeded on 24- well plates. Metabolic activity of cells was determined for seven consecutive days. Briefly, cultures were rinsed twice with PBS and the tetrazolium salt (MTT, Sigma, Germany) solution (0.5 mg/ml) was added to each well. After 3 h of incubation (37 °C) the resulting formazancrystals were dissolved in a dimethylsulfoxide (DMSO, POCH, Poland) solution. Absorbance at 570 nm (reference wavelength λ = 630 nm) measured by a UV/VIS spectrophotometer (CECIL CE 20121, Buck Scientific, USA) was used to determine UB-SMCs growth curve.

#### Analysis of cells growth in real time using the X-Celligence system

Cell growth was monitored also in real time using the X-Celligence system (Roche, Germany). Cells were seeded on 16- well plate (14 examined, two controls) (E- Plate 16, Roche, Germany) at a density of 2 × 10^4^cells/cm^2^. X-Celligence system base on a measurement of the impedance (changes in electrical resistance) of adherent cells via biosensors placed on the bottom of each well of the plate. Impedance is then processed to Cell Index. Measurements were made every 30 min. for a period of 7 days. Cell growth curve was plotted as a dependence of Cell Index from the time of culture.

### *β*-*Galactosidase assay*

Presence of aging cells in culture was assessed by determination of β-galactosidase activity using a Beta-Galactosidase Staining Kit (Clontech, USA). Tests were performed when the cells reached 70- 90 % confluence, just before the subsequent passages. Culture was washed twice with PBS, followed by addition of a fixing buffer. After 5–10 min. of incubation at room temperature, culture was again washed with PBS. Then the staining solution was added (X-Gal Staining Mix). After 12 h incubation, the percentage of β-galactosidase positive cells was determined by counting the number of blue cells under inverted microscope. As a control the mouse 3 T3 fibroblasts were used.

#### Statistical analysis

Data were analyzed by SPSS Statistica (SPSS, USA) using Kruskal Wallis and Mann–Whitney *U* tests. Bonferroni correction was used for pairwise comparisons. The statistical significance was deemed at *p* ≤ 0.05.

## Results

### Histological and immunohistochemical analysis of smooth muscle layer fragment

**Fig. 2:**
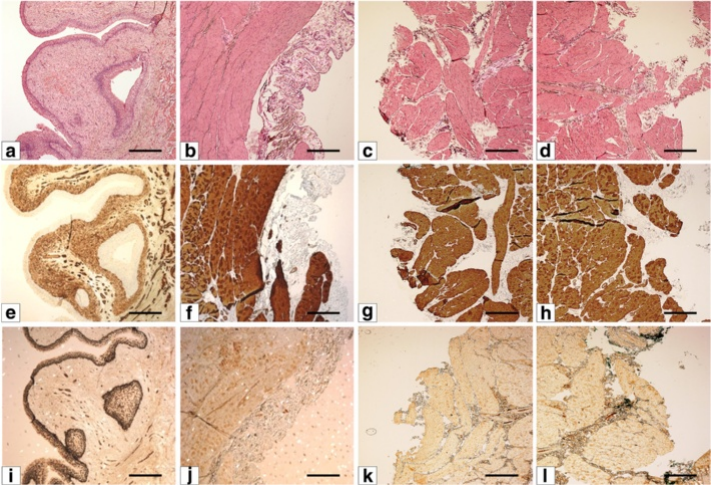
Histological and immunohistochemical staining of the bladder wall before (**a,b,e,f,i,j**) and after surgical removal of the mucosa/submucosa and serosa (**c,d,g,h,k,l**): hematoxylin and eosin (HE) staining (**a,b,c,d**), anti- α-smooth muscle actin (α-SMA) staining (**e,f,g,h**) and anti- p63 protein (p63) staining (**i,j,k,l**). Light microscope, objective magnification x4; bar 400μm

Histological and immunohistochemical staining of urinary bladder wall prior to removal of the mucosa/submucosa and serosa showed the presence of all layers characteristic for urinary bladder wall (Fig. 2a,b,e,f,i,j). Strong positive reactions for α-SMA and p63 were observed in detrusor muscle and urothelium, respectively (Fig. 2f,i). Histological and immunohistochemical analysis of the bladder wall after surgical removal of the mucosa/submucosa and serosa confirmed the presence of smooth muscle and absence of adjacent layers (Fig. 2c,d,g,h,k,l).

### Efficiency of the urinary bladder smooth muscle cell isolation

**Fig. 3:**
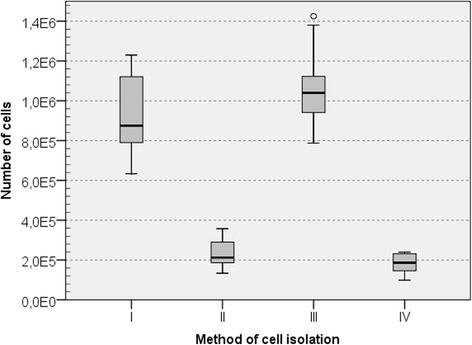
Number of cells isolated from 1 cm2 of urinary bladder muscle. Boxes indicate median and interqartile range with vertical lines depicting the range. I vs II *p* < 0.001; I vs III *p* = 1.00; I vs IV *p* < 0.001; II vs III *p* < 0.001; II vs IV *p* = 1.00; III vs IV *p* < 0.001

The average numbers of cells isolated using four enzymatic protocols was shown in Fig. 3. The methods utilizing collagenase in combination with dispase (method I) or collagenase only (method III) were more efficient than other two methods. The numbers of cells isolated from a 1cm^2^ smooth muscle layer for the method I or III was 5 times higher compared to methods II and IV.

### Cell morphology

**Fig. 4:**
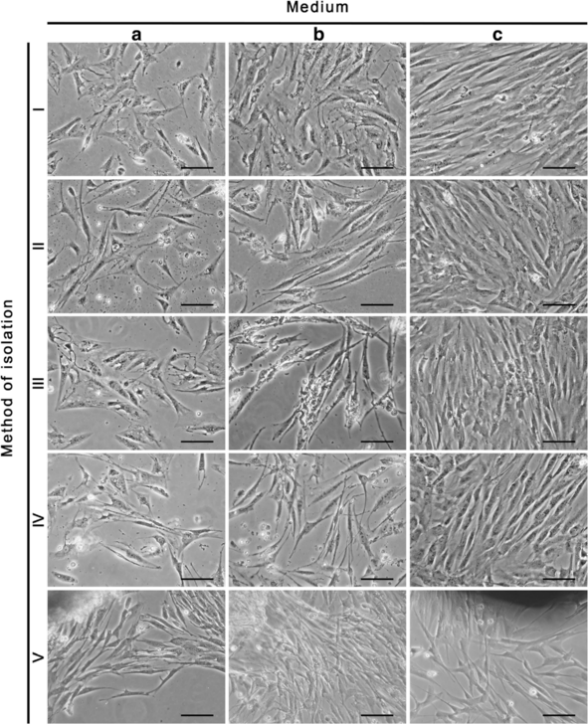
Bladder smooth muscle cells in the fifth day of culture. Cells isolated by methods I-V and cultured in three different media: DMEM HG with FBS Pan-Biotech (**a**), DMEM HG with FBS Sigma (**b**) and SmGM-2 (**c**). Inverted microscope, objective magnification x10; bar 100μm

Morphology of cells in the established primary cultures, differed significantly among the three media used (Fig. 4). The best results were observed when isolated cells were cultured in a SmGM-2 (C) (Fig. 4, ic, iic, iiic, ivc, vc). Cells in these cultures were elongated, spindle-shaped, bipolar with a centrally located nucleus and exhibited characteristic for the smooth muscle cells “hills and valleys” growth pattern. Intensive cell divisions allowed to reach confluence in 4–5 day of culture. Much less effective results were obtained with two other media (A, B). Primary cultures established in medium B (DMEM HG supplemented with FBS Sigma) in most cases were characterized by the presence of cells with morphology typical for smooth muscle cells. However, common cells detachments precluded the cultures from reaching confluence (Fig. 4, ib, iib, iiib, ivb, vb). When using the medium A (DMEM HG, FBS Pan-Biotech) cells detachment was not as frequent. However, morphological changes in cells, such as cytoplasm vacuolation and irregular cell shape were observed (Fig. 4, ia, iia, iiia, iva, va). Cell proliferation was inhibited and such as in the case of medium B the confluence was not reached.

Type of culture medium had no effect on morphology of cells obtained by explant culture, but often cells with different morphology were present in culture, demonstrating the co-culture. However, the type of medium have an impact on cell attachment to the surface of culture dishes. In the case of medium B (DMEM HG with FBS Sigma) and medium A (DMEM HG with FBS Pan-Biotech) cell detachment was observed. When cells were cultured in medium C (SmGM-2) they remained tightly attached to the dish surface.

### Success rate of established cell cultures

Data for success rate of establishment of primary cultures of porcine urinary bladder smooth muscle cells were presented in Table 2. All primary cultures established in medium C (SmGM-2) showed normal morphology, and increase in number of cells which allowed to reach confluence. Success rate of cultures grown in medium C regardless of method of isolation was 100% (36/36 cultures). In the case of all cultures established in medium B (DMEM HG, FBS Sigma) cells showed normal morphology, but common cell detachments have not allowed to reach a confluency, and consequently the success rate was 0%. Cultures established in medium A (DMEM HG, FBS Pan-Biotech) were characterized by changes in cellular morphology and inhibition of cell proliferation. However, slightly better cell adhesion compared to medium B allowed to achieve a confluency with normal morphology in 2 from 45 cultures (Table 2). Establishment of primary cultures of UB- SMCs using the explant technique was the least effective, and reproducible among the compared methods. Most of the tissue fragments detached from the surface of culture dish during the first days of culture, while the attached explants not always lead to cells spreading. Finally, none of the explants cultures was successful. Cells migrating out of explants formed colonies, but their quantity was too low to achieve confluence.

**Table 2:** Success rate of establishment of primary cultures of porcine urinary bladder smooth muscle cells

Isolation method	Growth medium	Number of confluent cultures	Number of cultures with normal morphology	Success rate
I	A	3/9 (33.3%)	2/9 (22.2%)	1/9 (11.1%)
	B	0/9 (0 %)	7/9 (77.8%)	0/9 (0 %)
	C	9/9 (100%)	9/9 (100%)	9/9 (100%)
II	A	2/9 (22.2%)	0/9 (0 %)	0/9 (0 %)
	B	0/9 (0 %)	0/9 (0 %)	0/9 (0 %)
	C	9/9 (100%)	9/9 (100%)	9/9 (100%)
III	A	3/9 (33.3%)	1/9 (11.1%)	1/9 (11.1%)
	B	0/9 (0 %)	5/9 (55.6%)	0/9 (0 %)
	C	9/9 (100%)	9/9 (100%)	9/9 (100%)
IV	A	0/9 (0 %)	1/9 (11.1%)	0/9 (0 %)
	B	0/9 (0 %)	0/9 (0 %)	0/9 (0 %)
	C	9/9 (100%)	9/9 (100%)	9/9(100%)
V	A	0/9 (0 %)	0/9 (0 %)	0/9 (0 %)
	B	0/9 (0 %)	0/9 (0 %)	0/9 (0 %)
	C	0/9 (0 %)	2/9 (22.2%)	0/9 (0 %)

### Cell phenotype

**Fig. 5:**
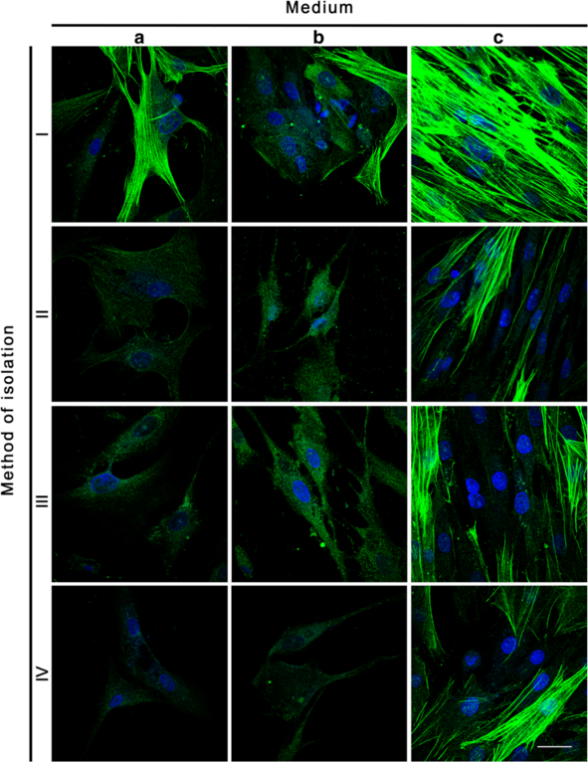
Expression of α-smooth muscle actin in primary cultures of cells isolated by the methods I-IV and cultured in three different media: DMEM HG with FBS Pan-Biotech (**a**), DMEM HG with FBS Sigma (**b**) and SmGM-2 (**c**). Immunofluorescence staining with antibody against α-smooth muscle actin, nuclei stained with DAPI, laser scanning confocal microscopy; bar 25μm

**Fig. 6:**
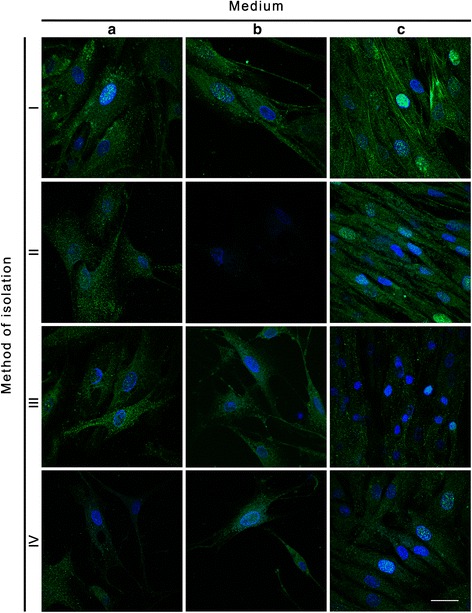
Expression of smoothelin in primary cultures of cells isolated by the methods I-IV and cultured in three different media: DMEM HG with FBS Pan-Biotech (**a**), DMEM HG with FBS Sigma (**b**) and SmGM-2 (**c**). Immunofluorescence staining with antibody against smoothelin, nuclei stained with DAPI, laser scanning confocal microscopy; bar 25μm

**Fig. 7:**
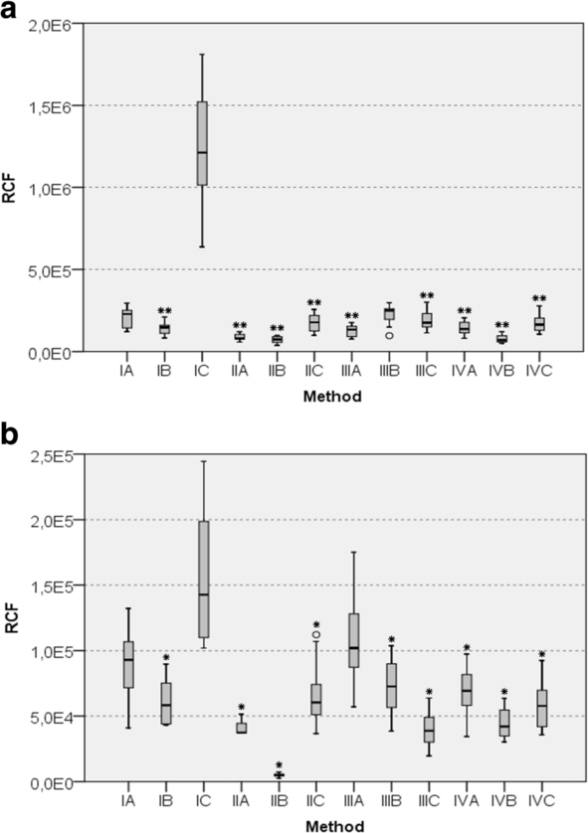
Relative cell fluorescence (RCF) for anti- smoothelin (**a**) and anti-α-SMA (**b**) in primary cultures of cells isolated by methods I-IV and cultured in three different media (**a-c**). Boxes indicate median and interqartile range with vertical lines depicting the range. * *p* < 0.001 vs IC; ** *p* < 0.05 vs IC

**Fig. 8:**
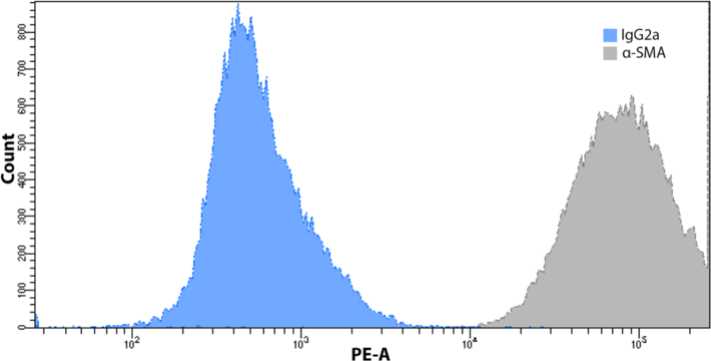
Flow cytometry analysis. Cells from fourth passage exhibit 99.5% expression of α-SMA. Isotype control using IgG2a excluded unspecific binding of antibody

Immunofluorescence stainings with anti α-SMA and anti- smoothelin allowed to analyze the phenotype of UB- SMCs and homogeneity of established cultures (Fig. 5, 6 and 7, Table 3). Ani- α-SMA staining revealed that the most phenotypically homogenous cultures of UB-SMCs were established when cells were isolated using method I and cultured in SmGM-2 (C) (Fig. 5, ic, Table 3). Cells in these cultures were rich in actin filaments, spindle-shaped and close, parallel located. Relative cell fluorescence (RCF) for α-SMA was significantly higher in these cultures compared to the others (*p* < 0.001) (Fig. 7a). Positive for α-SMA cells were also observed in all cultures with cells isolated by method I (independently from used medium) and cultured in SmGM-2 (independently from isolation method) and cultures with cells isolated by methods II and III and cultivated in DMEM HG with FBS Pan-Biotech (Table 3). However, only the cells cultured in medium C, retained morphology typical for UB- SMCs. In remaining methods, there were no cells exhibiting the presence of smooth muscle α-actin filaments (Fig. 5, ii a,b, iii b, iv b). Comparable results were obtained for anti- smoothelin staining. The strongest expression of smoothelin was observed in cells isolated using method I and cultured in SmGM-2 (Fig. 6, ic). Only in these cultures smoothelin formed filaments. RCF for smoothelin was significantly higher in these cultures compared to the others (*p* < 0.05) (Fig. 7b). In some cultures smoothelin protein was observed only in the foci form located in the nucleus (Fig. 6, ia, ib, iic, ivc) or cytoplasm (Fig. 6, iia, iiia, iiib, iiic, iva, ivb). Cells isolated by method II and cultured in medium B showed no presence of smoothelin (Fig. 6, iib; Fig. 7b). Additionally for cells isolated using method I and cultured in SmGM-2 cell phenotype was confirmed using flow cytometry. Only in this case proper number of cells necessary for flow cytometry analysis was achieved. Obtained results showed, that almost all isolated cells (99.7% ± 0.2) expressed the α-SMA (Fig. 8).

**Table 3:** Cell population homogeneity

Method		α-SMA [%]	Smoothelin [%]
I	A	31	0
	B	3	0
	C	98	83
II	A	0	0
	B	0	0
	C	29	0
III	A	11	0
	B	0	0
	C	54	0
IV	A	12	0
	B	0	0
	C	40	0

Percentage of cells showing α-smooth muscle actin (α-SMA) and smoothelin filaments organization

### Selection of the most efficient method of establishment of primary culture of urinary bladder smooth muscle cells

Comparison of all analyzed data (isolation efficiency, success rate of establishment of primary culture, cell phenotype) indicated that isolation of cells using combination of collagenase II and dispase II (method I) and culture in SmGM-2 (medium C) are the most effective and repetitive conditions for isolation and culture of porcine urinary bladder smooth muscle cells.

### Urinary bladder smooth muscle cell proliferation rate

**Fig. 9:**
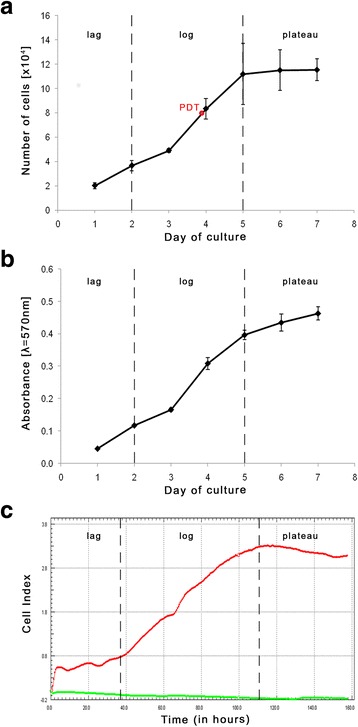
Porcine urinary bladder smooth-muscle cell growth curve plotted based on the number of cells in culture determined by trypan blue assay (**a**) or MTT assay (**b**) and measurements of impedance using the X-Celligence system; red curve - test samples (cultures of porcine bladder smooth muscle cells), green curve- control (medium SmGM-2) (**c**). Growth phases (log, lag, plateau) are separated by dotted lines, PDT - population doubling time

Analysis of cell growth curves (Fig. 9) allowed to observe the phases of growth characteristic for the adherent cell culture. Independently from the method of measurements, for the first 2 days of culture low proliferation rate related to slight increase in the number of cells corresponding to the adaptation phase (lag) was observed. From the second to fifth day rate of cell proliferation increased significantly, what was characteristic for the logarithmic growth phase (log). Population doubling time (PDT) was 1.87 day, which corresponds to approximately 4th day of culture of smooth muscle cells (Fig. 9a). With the fifth day culture growth inhibition was observed, associated with the beginning of the stationary phase (plateau).

### Aging of urinary bladder smooth muscle cells

**Fig. 10:**
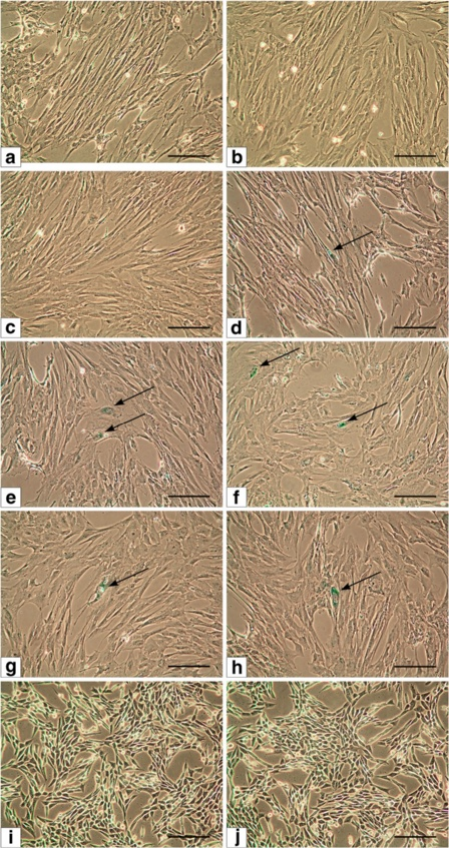
Analysis of cellular senescence in cultures of porcine urinary bladder smooth muscle cells from 1st to 8th passage (**a**-**h** respectively); negative control - mouse 3T3 fibroblasts (**i,j**); single aging cells indicated by arrows. Inverted microscope, objective magnification x10; bar 200μm

Presence of senescent cells characterized by increased β-galactosidase activity was not observed in cultures of 1st, 2nd and 3rd passage (Fig. 10a-c). First single ageing cells were found in cultures from the 4th passage (Fig. 10d). After subsequent passages the number of aging cells was unchanged (Fig. 10e-g). To the 8th passage only a single aging cells were observed (Fig. 10h). We found that from 1 cm^2^ of smooth muscle tissue biopsy, from 8 passages, we are able to obtain ~25 × 10^7^ UB- SMCs.

## Discussion

Despite several published UB-SMCs isolation and culture protocols doubts still remain about which one is optimal for clinical application. In this study, we compared isolation and expansion techniques of UB-SMCs to select the most repeatable and efficient for tissue engineering applications.

As a starting material, we used porcine urinary bladders because they are closely related to human in terms of their anatomy and physiology and often are the model of choice for urinary bladder reconstruction in the preclinical stage [15–17]. Sufficiently large size of porcine urinary bladder allowed to compare all methods of isolations using the material from the same donor and to exclude the inter individual variability. In order to be able to compare earlier presented protocols [9–12] we standardized tissue size, volume of digestion solution and seeding density of cells.

Histological and immunohistochemical stainings confirmed that the process of surgical removal of serosa and urothelium was performed properly and fragments of urinary bladder smooth muscles were free from adjacent tissues. However, the most of established cultures were heterogeneous. This phenomenon was the most visible when the primary cultures were established by the explant technique. The fibroblasts present in these cultures could came from the remains of the serosa and submucosa or connective tissue surrounding the muscle fibers. Although, the low purity of UB- SMCs is not restricted to cultures established by explant technique only and is a common problem of numerous enzymatic methods, what was previously reported by other authors [18]. Contamination of UB- SMCs primary culture with fibroblasts is very unfavorable because they can overgrowth the primary culture. In this study, the most homogenous culture (98% purity) was obtained when UB-SMCs were isolated with collagenase and dispase and cultured in SmGM-2.

Two enzymatic methods utilizing collagenase and dispase (method I) and collagenase only (method III) allowed to isolate significantly higher number of viable cells compared to the others. Using these methods we were able to isolate ~1x10^6^ cells from 1 cm^2^ of smooth muscle. The other two methods (II and IV) utilizing collagenase and trypsin allowed to obtain five time less number of cells. A possible explanation of this phenomenon is that trypsin digest tissue more aggressively than collagenase and dispase therefore digestion with trypsin led to cell destroying and consequently low isolation yield. Comparison of isolation results obtained in this study with original protocols [9–12] is impossible as no data on number of isolated cells were published. However, the numbers of cells isolated using methods I and III were significantly higher compared to other published previously protocols [19].

In the case of establishment of primary culture using the tissue explant technique one of the most critical step was attachment of tissue pieces to the bottom of a culture dish. Most of tissue explants detached from the culture surface during the first days of culture and consequently it was the least effective, and reproducible among the compared methods. The most difficult was attachment of a tissue explants onto a glass coverslip for immunofluorescence analysis, therefore we were not able to analyze the α-SMA and smoothelin expression in the primary culture established by this method.

Analysis of cell morphology revealed that the most efficient medium for establishment of UB-SMCs was SmGM-2. Only cells cultured in SmGM-2 exhibited characteristic for the smooth muscle cells “hills and valleys” growth pattern. These observations contradict previously published results [9–12] which indicated that smooth muscle cells cultured in RPMI-1640 or DMEM/ Ham’s F-12 supplemented with FBS exhibit “hills and valleys” growth pattern.

Immunofluorescence staining showed that both isolation method and growth medium have strong impact on the phenotype of cells in culture. Cells isolated by method I (independently on culture medium) or cultured in SmGM-2 (independently on isolation method) express α-SMA. The strongest expression of α-SMA was observed when cells were isolated by the method I and cultured in SmGM-2. In this case the cultures were also most homogenous. This result was confirmed also by flow cytometry, which confirmed that 99.7 % of isolated cells expressed α-SMA. This analysis revealed that although the numbers of cells isolated by methods I and III are comparable, only the method I allowed to isolate UB-SMCs. These results contradict those obtained by Ma et al. who indicated that 99 % of cells isolated by method IV expressed α-SMA and myosin [12]. Because, α-SMA is a contractile protein present in both smooth muscle cells and myofibroblasts [20] we used additional marker- smoothelin to differentiate myofybroblasts from the smooth muscle cells. Smoothelin is a marker of highly differentiated and contractile SMCs [21]. The highest level of smoothelin was observed in cells isolated by method I and cultured in SmGM-2. Expression of smoothelin in UB- SMCs is controversial because this protein is not expressed spontaneously in cultured cells. In the primary culture of SMCs using the tissue explant technique the smoothelin mRNA decreased to undetectable levels within 12h after dissection [22, 23]. No expression of smoothelin was detected in smooth muscle cells isolated from urinary bladders, aorta and esophagus [24]. To our best knowledge, it is the first report of positive expression of smoothelin in primary culture of UB-SMCs. This observation has strong implications for tissue engineering, because creation of the urinary bladder wall de novo from functional SMCs could accelerate restoring of the urinary bladder function.

The fundamental question of any cell therapy is to know how many times the primary culture can be passaged before cell implantation. To obtain a high number of cells the primary culture has to be passaged many times. However, senescence decrease regenerative potential of cells therefore it should be analyze before any cell implantation. In this study we indicated that UB-SMCs can be cultured up to 8th passage without significant signs of senescence. Population doubling time (PDT) for UB-SMCs was 1.87 days (44.9h) and was shorter compared to results obtained by Huber et al., where the PDT of UB-SMCs cells was 5.3 days (128.2h) [24]. These results confirm that isolated in this study UB-SMCs had high proliferative activity. We found that from 1 cm^2^ of smooth muscle we are able to obtain ~25 × 10^7^ UB-SMCs in 5 weeks of culture. This protocol enabled us to obtain large, clinically relevant numbers of UB-SMCs which can be used for creation of the urinary bladder wall de novo utilizing tissue engineering methodologies.

## Conclusions

This study indicated that success of establishment of primary culture of urinary bladder smooth muscle cells is critically dependent on both- method of cell isolation and media used for cell culture. The most efficient method of establishment of UB-SMCs culture is enzymatic digestion of urinary bladder tissue in collagenase II and dispase II and culture of isolated cells in SmGM-2. This is an efficient and repetitive method for obtaining high number of good quality UB-SMCs for tissue engineering.
